# Coronavirus disease 2019 (COVID-19) pathogenesis: a concise narrative review

**DOI:** 10.11604/pamj.2021.39.8.23546

**Published:** 2021-05-03

**Authors:** Hamza Elhamzaoui, Houssam Rebahi, Abdelhamid Hachimi

**Affiliations:** 1 Critical Care Department, Mohammed VIth University Hospital of Marrakech, Cadi Ayyad University, Marrakech, Morocco

**Keywords:** Coronavirus, SARS-CoV-2, COVID-19, pathogenesis

## Abstract

SARS-CoV-2 is the third zoonotic coronavirus. Since December 2019, it has spread through the globe and infects more than four million patients (as of May 10^th^, 2020). The disease was named coronavirus disease 2019 (COVID-19) by the World Health Organization (WHO). It involves many organs and systems in the human organism. We aimed to describe the pathogenesis of the COVID-19.

## Introduction

Of the coronaviridae family, human coronaviruses (HCoVs) include six strains. The first HCoV, named B814, was identified in 1965 in nasal swabs in patients with cold [1]. Since then, more HCoVs have been isolated: HCoV-229E (229E), HCoV-OC43 (OC43), HCoV-NL63 (NL63), HCoV-HKU1 (HKU1), severe acute respiratory syndrome coronavirus (SARS-CoV-2) and Middle East respiratory syndrome coronavirus (MERS-CoV) [2]. They cause primarily upper respiratory and gastrointestinal infections, which vary from mild (common cold) to severe acute respiratory syndrome with possible multi-organ failure [3]. SARS-CoV discovered during the SARS epidemic, started from Guangdong province in Southern China in November 2002 and spread to other countries in Asia, North America and Europe [4,5]. While, since 2012, MERS-CoV is responsible for a progressing outbreak in the Middle East [6]. After SARS-CoV and MERS-CoV, the third zoonotic coronavirus, novel coronavirus (2019-nCoV) has spread and infected more than four million persons worldwide (as of May 10^th^, 2020), since December 2019, from Wuhan, China [7]. The International Committee on Taxonomy of Viruses named 2019-nCoV as severe acute respiratory syndrome coronavirus 2 (SARS-CoV-2). The World Health Organization titled the disease caused by SARS-CoV-2 as coronavirus disease 2019 (COVID-19) [8-10]. Chan *et al*. Hui *et al*. Zhou *et al*. and Wu *et al*. suggested that the origin of SARS-CoV-2 was bats but the intermediate host remains unknown [11-14].

Since the first case, COVID-19 could be qualified as a “great imitator” because it is not a simple lung disease. It can cause more severe impairment such as heart rhythm problems, heart failure, acute kidney failure, hemorrhagic stroke, seizures, meningitis Guillain-Barre syndrome, or thromboembolic events [15,16]. When the virus gains the body via eyes, nose, or mouth, it invades cells by connecting to ACE2 receptors, which are found in the organs. In this review, we aimed to set out SARS-CoV-2 pathogenesis.

## Methods

We performed a literature search in multiple databases dedicated to the literature about COVID-19: NEJM database [17], ESICM database [18], Elsevier information center [19], and Medscape center [20]. The keywords used in the research were coronavirus, SARS, MERS, SARS-CoV-2, MERS-CoV, SARS-CoV-1, SARS-CoV-2, 2019-nCoV and COVID-19.

## Current status of knowledge

**Genetic predispositions:** several factors make some people susceptible to the coronavirus and present phenotypes ranging from asymptomatic to severe forms of COVID-19 with acute respiratory distress syndrome with possible multi-organ failure. Gralinski *et al*. suggested that the alteration of the function of the innate-immune modulatory gene Ticam2 makes rodents highly susceptible to the coronavirus. This gene, Ticam2, codes for a helper protein in the activation of a family of receptors (TLR, for toll-like receptor) involved in the mechanisms of innate immunity [21]. Zhao *et al*. highlighted the second component [22]. They showed that blood group A is linked to a higher risk of acquiring COVID-19 (OR 1.279; 95% CI 1.136-1.440; p<0.001) and death (OR 1.482; 95%CI 1.113-1.972; p=0.008,) compared with other blood groups. Cheng *et al*. expressed the same findings for SARS-CoV-2 in 2005 [23]. The third genetic component points out the relationship between human leukocyte antigen (HLA) and the risk to contract and the severity of COVID-19. Indeed, the susceptibility to SARS-CoV-2 is associated with HLA-B*46: 01 [24], as previously expressed by Lin *et al*. for SARS-CoV-2 [25].

The fourth component is related to the angiotensin-converting enzyme 2 (ACE2). According to Li *et al*. ACE2 is the functional receptor of SARS-CoV-2 [26]. Effectively, Hamming *et al*. reported that this receptor is plenteous in the human epithelia of the lung and small intestine [27], with its presence in the vascular endothelium. Experimentally, as outlined by Kuba *et al*. [28], the infected ACE2 knockout mice are resistant to virus infection and the virus titers are 10^5^ fold lower than those isolated from the lung of SARS-CoV-2 infected wild-type mice, without evidence of inflammation in the lung histology. Recently, Yan *et al*. [29], Zhang *et al*. [30] and Wan *et al*. [31] stated that ACE2 is involved in the pathogenesis of SARS-CoV-2. To find out more about the genes that influence SARS-CoV-2 infection, some projects are underway [32,33].

**Figure 1:**
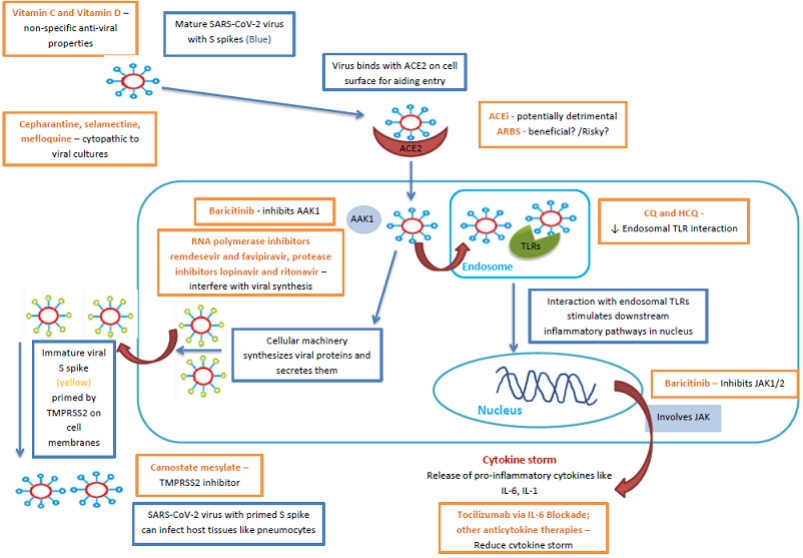
potential therapeutic targets for SARS-CoV-2 and COVID-19

**Pulmonary involvement:** angiotensin-converting enzyme 2 (ACE2) is the host receptor through which SARS-CoV-2 enters cells (Figure 1) [34]. In normal lung tissue, ACE2 is mainly expressed by type I and type II alveolar epithelial cells. It was reported that 83% of type II alveolar cells expressed ACE2. Therefore, COVID-19 infection causes damages to most type II alveolar cells. After alveolar cell injury, transforming growth factor-β (TGF-β) is released in the tissue to promote lung repair. Virus infection often leads to excessive activation of the TGF-β pathway, which leads to the occurrence of pulmonary fibrosis [35,36]. In the early phases of the lung pathology of COVID-19, Tian *et al*. described findings of edema, proteinaceous exudate, focal reactive hyperplasia of pneumocytes with patchy inflammatory cellular infiltration and multinucleated giant cells in a pathologic examination of infected lungs [37]. Even though the COVID-19 meets the acute respiratory distress syndrome (ARDS) Berlin definition [38], it is a disease with distinctive phenotypes and can even be part of the ARDS mimics described by Aublanc *et al*. [39]. Its main characteristic is the dissociation between the severity of the hypoxemia and the maintenance of relatively good respiratory mechanics [40]. To conceptualize this phenomenon, Gattinoni L *et al*. [41] hypothesized a sequence of events: SARS-CoV-2 infection causes a modest local subpleural interstitial edema located between lung structures with different elastic properties [42]. The normal response is to increase minute ventilation, by increasing the tidal volume [43], which is associated with a more negative intrathoracic inspiratory pressure. However, the near-normal compliance explains why some of the patients present without dyspnea as the patient inhales the volume he expects.

Two peculiar phenotypes can be distinguished: type L, characterized by Low elastance (i.e. high compliance), low ventilation to perfusion ratio, low lung weight and low recruitability. These patients may remain unchanging for a period and then improve or worsen. The possible feature that can determine the evolution of the disease, other than severity, is the depth of the negative intrathoracic pressure associated with the increased tidal volume in spontaneous breathing. The increased lung permeability due to inflammation, associated with this negative intrathoracic pressure, causes interstitial lung edema. As the edema increases, the lung weight increases, until eventually the gas volume in the lung decreases and the tidal volumes decrease. This phenomenon leads to a transition from type L, to type H, which is characterized by high elastance, high right-to-left shunt, high lung weight and high recruitability. These patients likely develop self-inflicted ventilator-induced lung injury [40,41]. These different COVID-19 patterns depend on the interaction between three factors [41]: 1) the severity of the infection, the host response, and comorbidities; 2) the ventilatory response to hypoxemia and 3) the time between the beginning of the disease and the hospitalization.

**Figure 2:**
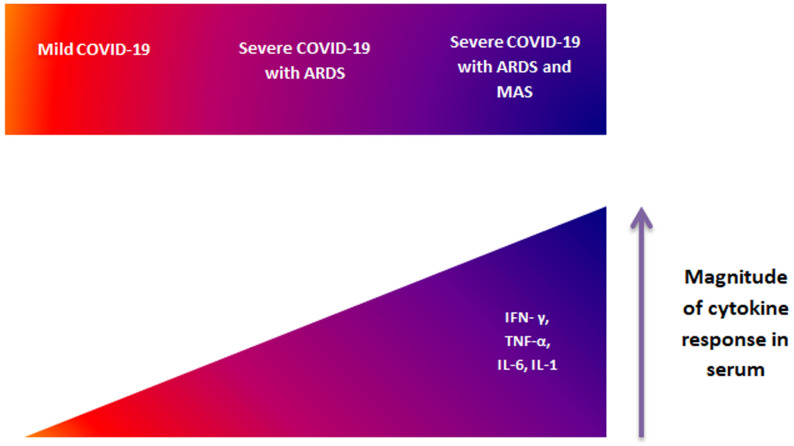
hyper-cytokinemia accompanying the overlap between acute respiratory distress syndrome (ARDS) and macrophage activation syndrome (MAS) associated with COVID-19

**Hematological disorders:** besides, in the early phases of infection, ACE-2 consumption by viral entry is predicted to increase local angiotensin II concentration. Among the known effects of angiotensin II are vasoconstriction, endothelial activation, and pro-inflammatory cytokine release [44]. Consequently, viral injury, disordered cytokine release and platelet activation by angiotensin II induce localized microvascular inflammation, which triggers endothelial activation, leading to vasodilation and pro-thrombotic conditions. The procoagulant state has also long been recognized as part of ARDS pathophysiology, demonstrated by the identification of diffuse pulmonary endothelial injury associated with platelets' activation, macro- and micro-thrombi thought to be either embolic, formed in situ, or both [45]. Moreover, activated platelets, neutrophils, endothelial cells, neutrophil extracellular traps, microparticles, and coagulation proteases in ARDS have been associated with the process of deep vein thrombosis formation [46]. This explains the high prevalence of acute pulmonary embolism recently reported in COVID-19 patients admitted for hypoxemic acute respiratory failure [47]. Accumulating evidence suggests that SARS-CoV-2 might induce a cytokine storm (Figure 2) [48]. Patients with severe pneumonia or ARDS develop systemic manifestations of hyper inflammation called secondary hemophagocytic lymphocytosis (sHLH), also called macrophage activation syndrome (MAS) [49]. This hyperinflammation is characterized by an increased release of cytokines, particularly interleukin (IL)-6, IL-1 by binding to ACE-2 in the lung tissue, IL-6, interferon-γ and tumor necrosis factor-α [50]. This exaggerated response is responsible for significant tissue damage that could lead to multiorgan failure. Multiple studies comparing severe and moderate forms of COVID-19 concluded that the cytokine storm is associated with the severity of the disease [51,52].

**Figure 3:**
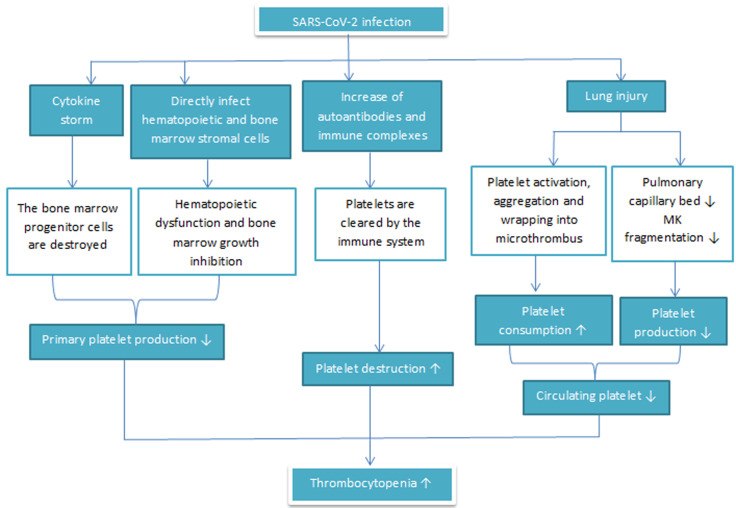
the possible mechanisms of thrombocytopenia in COVID-19 patients

Many studies reported thrombocytopenia in patients with COVID-19, but its mechanism is still unclear. Xu *et al*. [53] described the hematological changes of thrombocytopenia in patients with COVID-19 and proposed three possible mechanisms (Figure 3) [53] by which SARS-CoV-2 can induce thrombocytopenia: a) SARS-CoV-2 can reduce platelet production by direct infection of bone marrow cells and therefore inhibit platelet synthesis. The virus can also do so through the cytokine storm that destroys bone marrow progenitor cells; b) COVID-19 infection can increase autoantibodies and immune complexes resulting in the specific destruction of platelets by the immune system; c) and damages of the lung tissues and pulmonary endothelial cells by the SARS-CoV-2 infection activate platelets in the lungs, resulting in aggregation and formation of microthrombi, which leads to an increase in platelet consumption.

**Cardiovascular involvement:** the cardiovascular (CV) system can be involved in different ways. The common mechanisms responsible for cardiovascular complications in COVID-19 are: a) direct myocardial injury [54]: SARS-CoV-2 enters human cells by binding to angiotensin-converting enzyme 2 (ACE2), a membrane-bound aminopeptidase that is highly expressed in the heart and lungs. ACE2 plays an important role in the neurohumoral regulation of the CV system under normal health conditions as well as in various diseases. Binding between the SARS-CoV-2 and ACE2 can lead to alterations in ACE2 signaling pathways, leading to acute damage to the myocardium and lungs; b) systemic inflammation [54]: the most severe forms of COVID-19 are characterized by an acute systemic inflammatory response and cytokine storm, which can lead to multi-organ injury, leading to organ failure. Studies have shown elevated circular levels of pro-inflammatory cytokines in patients with critical COVID-19; c) altered myocardial supply-demand relationship [55]: the increased metabolic demand associated with systemic infection coupled with hypoxia caused by the acute respiratory disease may alter the relationship between myocardial oxygen supply and demand and result in acute myocardial injury; d) plaque rupture and coronary thrombosis [56]: systemic inflammation and increased shear stress due to increased coronary blood flow may precipitate plaque rupture and result in acute myocardial infarction. The prothrombotic appearance created by systemic inflammation further increases the risk; e) adverse effects of various therapies [57]: various antiviral drugs, corticosteroids, and other therapies to treat the COVID-19 channel also have deleterious effects on the CV system. Electrolyte imbalances - electrolyte imbalances can occur in any critical systemic disease and precipitate arrhythmias, particularly in hospitalized patients with underlying heart disease. Of particular concern is the hypokalemia of COVID-19 due to the interaction of SARS-CoV-2 with the renin-angiotensin-aldosterone system, which increases susceptibility to various cardiac arrhythmias.

Role of underlying CV co-morbidities patients with the pre-existing cardiovascular disease appear to have an increased susceptibility to the development of COVID-19 and tend to have more severe disease with poorer clinical outcomes. Although the prevalence of diabetes and hypertension in this cohort is the same as in the general Chinese population, the prevalence of the cardiovascular disease is significantly higher. More importantly, the presence of diabetes, CVD and hypertension were associated with a two-fold, three-fold and two-fold increased risk of serious illness or requiring admission to an intensive care unit (ICU), suggesting a prognostic impact of these co-morbidities [57].

**Acute kidney injury:** the acute kidney injury (AKI) is an independent mortality factor, in SARS-CoV-2 infection, with an OR of 4.057 (95% CI 1.46-11.27; p<0.001) [58]. SARS-CoV-2-related AKI is a serious complication in 0.5 to 29% of hospitalized patients, particularly in the ICU setting [59-62], due to various etiology such as hemodynamic and cardiac disorders, invasive mechanical ventilation with impaired gas exchange, and occurrence of nosocomial sepsis; in addition to inflammatory substances release, microcirculatory alteration, and tubular injury [63]. Other factors are risks for AKI in acute respiratory distress syndrome (ARDS) including age, the severity of the illness, diabetes, obesity, and the history of heart failure [64]. Furthermore, the abundance, in the kidney tubular cells, of ACE2 as receptor facilitates the entry into target cells [26,27]; this infection participates in the worsening of the local inflammation, thus the incidence and the duration of AKI episodes [65]. In a postmortem renal histopathological analysis, Su *et al*. noted the invasion of SARS-CoV-2 into kidney parenchyma with diffuse proximal tubule injury, non-isometric vacuolar degeneration and even frank necrosis was observed, prominent erythrocyte aggregates obstructing the lumen of capillaries without platelet or fibrinoid material, without vasculitis, interstitial inflammation or hemorrhage, by light microscopy. The electron microscopic study described clusters of coronavirus in the tubular epithelium and podocytes. Additionally, ACE2 receptors were upregulated, and immunostaining with SARS-CoV-2 nucleoprotein antibody was positive in tubules [66]. Summarily, three mechanisms are involved in the SARS-CoV-2 associated AKI: cytokine damage, lung, and heart interactions and systemic effects [67] without forgetting direct lesions of the virus.

**Neurological involvement:** in the immunocompetent host, viral brain infections are rare, in general. But, neurotropic viruses can infect the central nervous system (CNS) (encephalitis, meningitis, myelitis, strokes) in the presence of temporary immunosuppression. The peripheral nervous system can be also affected (neuropathy, Guillain-Barré syndrome) [68]. The neurological complications during epidemic SARS-CoV-2 were not well studied. However, some case reports were presented with axonal peripheral neuropathy, myopathy with elevated creatinine kinase, or stroke [69-71]. For COVID-19, neurologic presentations were 36.4% of cases, more declared in severe patients (45.5% vs. 30.2% in non-severe patients). They can be subdivided into three groups: central nervous system manifestations (dizziness, headache, coma, acute cerebrovascular disease (intracerebral hemorrhage, hemorrhagic necrotizing encephalopathy, ischemic stroke), ataxia, acute myelitis, and seizure), peripheral nervous system manifestations (taste impairment, smell impairment, vision impairment, cranial neuropathies, Guillain-Barré Syndrome, and nerve pain) and skeletal muscle injury manifestations [72-78].

It is known that SARS-CoV-2 can induce neurological disease [79]. Effectively, Xu *et al*. mentioned the evident presence of SARS-CoV-2 in the brain of infected patients from the culture of the brain suspension [80]. Moreover, ACE2 receptors exist in the brainstem and the regions responsible for the regulation of cardiovascular function and were found in both neurons and glia [81,82]. Additionally, Yu *et al*. showed that SARS-CoV-2 belongs to the beta-coronavirus subgroup including MERS-CoV and SARS-CoV and has a high homological sequence with SARS-CoV-2 in the genomic analysis [83]. Given that, SARS-CoV-2 could have a potential neuroinvasion [84].

Regarding the route of invasion, first, the hematological and the lymphatic appear to be implausible because of the absence of virus particles in the non-neuronal cells in the infected brain areas [80,85,86]. Second, the synapse-connected route is more pointed out as possible via peripheral nerve terminals [87-90]. Third, since the last route has been described in other CoVs and avian bronchitis virus [87,89-92], there are case reports about smell or taste disturbances in the early stage of the disease [72]. Fourth, systemic inflammatory storm damaged the blood-brain-barrier and therefore sustained neuroinflammation [93].

**Hepatic damage:** Huang *et al*. [35] announced that 62% of critically ill patients had elevated aspartate aminotransferase (AST). Moreover, in a cohort of 1099 patients, Guan *et al*. observed that severe patients had more abnormal aminotransferase levels compared to non-severe [59]. In patients with COVID-19, the virus can directly affect the liver because about 2-10% of patients with COVID-19 develop diarrhea with SARS-CoV-2 RNA in stool and blood [94]. Liver disease is likely to be multifactorial and requires close biological monitoring to anticipate any progression. First, given that ACE2 receptors are expressed in cholangiocytes, SARS-CoV-2 may fix directly to cholangiocytes [95] and causes abnormal liver tests. Second, immune-mediated inflammation such as cytokine storm and hypoxia may also contribute to liver damage in critically ill patients with COVID-19. Third, there is a possible role of hepatotoxic drugs, especially lopinavir and ritonavir. Fourth, ventilation of severe recruitable ARDS with high levels of positive expiratory pressure may contribute to hepatic congestion [96]. Finally, in the histological examination, Cai *et al*. showed vesicular steatosis and degeneration of hepatocytes in the interlobular region and the hepatic sinuses and inflammatory cells in the hepatic sinuses [97]. However, no viral inclusions have been detected [98].

## Conclusion

COVID-19 is a “great imitator”. It primarily infects the lung and may invade the cardiovascular system, central and peripheral nervous systems, kidneys, and liver. Thus, the management is multidisciplinary including ventilator and hemodynamic supports, pharmacologic treatment, and specific technics such as plasma exchange, stem cells convalescent plasma.

### What is known about this topic

- SARS-CoV-2 is an emerging infection;
- SARS-CoV-2 affects essentially the pulmonary tract;
- Other organs might be impaired.

### What this study adds

- SARS-CoV-2 could cause multiorgan impairment;
- The initial presentation is sometimes unusual;
- The multiorgan involvement requires multidisciplinary management.
